# microRNA and the Post-Transcriptional Response to Oxidative Stress during Neuronal Differentiation: Implications for Neurodevelopmental and Psychiatric Disorders

**DOI:** 10.3390/life14050562

**Published:** 2024-04-26

**Authors:** Behnaz Khavari, Michelle M. Barnett, Ebrahim Mahmoudi, Michael P. Geaghan, Adam Graham, Murray J. Cairns

**Affiliations:** 1 School of Biomedical Sciences and Pharmacy, The University of Newcastle, Callaghan, NSW 2308, Australia; behnaz.khavari@uon.edu.au (B.K.); michelle.barnett@uon.edu.au (M.M.B.);; 2 Precision Medicine Research Program, Hunter Medical Research Institute, New Lambton Heights, NSW 2305, Australia

**Keywords:** oxidative stress, miRNA, psychiatric disorders, neurodevelopment, immune system, miR-137, miR-181b, DLK1-DIO3

## Abstract

Oxidative stress is one of the most important environmental exposures associated with psychiatric disorders, but the underlying molecular mechanisms remain to be elucidated. In a previous study, we observed a substantial alteration of the gene expression landscape in neuron-like cells that were differentiated from SH-SY5Y cells after or during exposure to oxidative stress, with a subset of dysregulated genes being enriched for neurodevelopmental processes. To further explore the regulatory mechanisms that might account for such profound perturbations, we have now applied small RNA-sequencing to investigate changes in the expression of miRNAs. These molecules are known to play crucial roles in brain development and response to stress through their capacity to suppress gene expression and influence complex biological networks. Through these analyses, we observed more than a hundred differentially expressed miRNAs, including 80 previously reported to be dysregulated in psychiatric disorders. The seven most influential miRNAs associated with pre-treatment exposure, including miR-138-5p, miR-96-5p, miR-34c-5p, miR-1287-5p, miR-497-5p, miR-195-5p, and miR-16-5p, supported by at least 10 negatively correlated mRNA connections, formed hubs in the interaction network with 134 genes enriched with neurobiological function, whereas in the co-treatment condition, miRNA-mRNA interaction pairs were enriched in cardiovascular and immunity-related disease ontologies. Interestingly, 12 differentially expressed miRNAs originated from the DLK1-DIO3 location, which encodes a schizophrenia-associated miRNA signature. Collectively, our findings suggest that early exposure to oxidative stress, before and during prenatal neuronal differentiation, might increase the risk of mental illnesses in adulthood by disturbing the expression of miRNAs that regulate neurodevelopmentally significant genes and networks.

## 1. Introduction

Oxidative stress, defined as elevated levels of reactive oxygen species (ROS), is thought to be one of the most important environmental exposures associated with many diseases, such as cancer, diabetes, cardiovascular, immunological, and neurological diseases, to name a few [1]. Physiological levels of ROS are crucial for key cellular processes since they act as second messengers to regulate signalling pathways. In the nervous system, in particular, ROS and redox states modulate neural fate by contributing to fundamental stages of neurodevelopment, such as neurogenesis, as well as the polarisation and maturation of neurons [1]. The brain is known to be particularly vulnerable to excessive ROS levels, which can adversely affect memory, learning, and cognition [2] and is suggested to be associated with psychiatric disorders. This is supported by elevated protein and lipid oxidation in the postmortem brain, cerebrospinal fluid (CSF), and blood of schizophrenia (SZ) cases and may be due to changes in antioxidant levels observed in patients with schizophrenia. These include reduced levels of plasma glutathione (GSH), catalase, and vitamins C and E. Accordingly, reduced GSH and superoxide dismutase 1 (SOD1) have also been observed in postmortem brain tissue and CSF from cases with the disorder [3]. Decreased activity and/or levels of antioxidant enzymes have also been reported in individuals with bipolar disorder (BD) [4] and autism spectrum disorder (ASD) [5].

Several layers of evidence suggest that oxidative stress might play a causal role in the development of psychiatric disorders. This includes the involvement of redox-sensitive proteins in neurogenesis and neuronal differentiation [6]. There are also polymorphisms in redox genes, such as the *GSH* system [7] and glutathione-S-transferase (*GST*) genes [8], which have been associated with schizophrenia and other psychiatric disorders [9]. Additionally, some known schizophrenia risk genes, including *DTNBP1*, *PRODH*, *NRG1*, *G72*, and *DISC1*, are directly involved in redox system function [3]. Given these oxidative stress-associated changes, it is reasonable to imagine that even subtle disturbances in the redox balance during brain development could negatively affect several important signalling pathways [6]. This is supported by animal models of psychiatric disorders, which suggest that GSH deficits and oxidative stress during brain development can lead to psychosis-like behaviour [3]. For example, a chronic brain deficit in GSH was observed to cause behavioural and cognitive anomalies in mice associated with schizophrenia and BD [10]. Even postnatal transient GSH deficiency during the development of the brain resulted in juvenile and adult rats showing some of the cognitive impairment and olfactory discrimination reported for schizophrenia [11].

How prenatal exposures to non-cytotoxic levels of oxidative stress affect critical neurodevelopmental processes, such as neural differentiation, and confer the risk of psychiatric disorders in adulthood is currently unknown. To explore these mechanisms, we recently investigated gene expression in neuron-like SH-SY5Y cells exposed to oxidative stress, both before and during differentiation, and observed substantial changes across the transcriptome [12]. These were predicted to induce large-scale perturbations in pathways associated with neurodevelopment and psychiatric disorders, suggesting that broad-reaching regulatory mechanisms such as post-transcriptional gene silencing might be coordinating the response to this exposure.

miRNAs are a class of small non-coding RNAs that coordinate an extended network of target genes at both the transcriptional and post-transcriptional levels. In most cases, these molecules bind to their target sequences at the miRNA recognition element (MRE) on the 3′-UTR of mature mRNAs through their 5′-end complementary sequence, known as the seed region. However, there are reports of miRNA binding sites detected in other regions of mRNA as well, such as the 5′ UTR, promoter, and coding region [13]. Since partial complementarity/homology suffices for this binding and activation of miRNA regulatory function, each miRNA is able to simultaneously suppress the expression of hundreds of genes post-transcriptionally by triggering their mRNA degradation or translation repression [14]. miRNAs’ expression is tissue- and developmental-specific, showing complicated temporospatial patterns in mammalian brains that are known to be critically involved in neurodevelopmental processes [15]. Unsurprisingly, a large number of genetic associations as well as expression studies suggest their dysregulation in various psychiatric disorders [14,16,17].

Given the implication of miRNAs in brain response and adaptation to environmental stress [18], we hypothesise that the rapid and widespread dysregulation of gene expression observed in oxidative stress-exposed cells before or during neuronal differentiation might be mediated to some extent by changes in miRNA expressions. Small RNA-sequencing on the same samples of SH-SY5Y cells exposed to oxidative stress, previously analysed by total RNA-sequencing [12], revealed the differential expression of more than a hundred miRNAs and their potential direct targeting of a subset of differentially expressed genes that were involved in various neuronal processes and both neurodevelopmental and immunity-related diseases.

## 2. Materials and Methods

### 2.1. Cell Culture and Differentiation

The human neuroblastoma SH-SY5Y cells were grown at a density of 20,000 cells/cm^2^ into 6-well plates, containing Dulbecco’s Modified Eagle’s Medium (DMEM, Irvine, UK, Sigma-Aldrich) cell culture medium, which was supplemented with 10% foetal bovine serum (FBS, Melbourne, VIC, Australia, Bovogen Biologicals), 2mM glutamine (Logan, UT, USA, HyClone), and 20 mM HEPES (New York, NY, USA, Thermofisher), and maintained in a 5% CO_2_ atmosphere at 37 °C. After 24 h, all-trans retinoic acid (ATRA, Sigma-Aldrich, St. Louis, MO, USA) was added to the medium at a final concentration of 10 µM to induce neuronal differentiation. The differentiation protocol lasted for 7 days, during which the cells were protected from light and the ATRA-supplemented medium was refreshed on the 3rd day. Successful differentiation was confirmed by observing neurite outgrowth as well as the expression of neuronal marker genes.

### 2.2. Application of Oxidative Stress

As described in detail previously [12], hydrogen peroxide (H_2_O_2_, Sigma-Aldrich, St. Louis, MO, USA) at a final concentration of 10 µM was applied through two different protocols to induce chronic oxidative stress in neuroblasts. In the co-treatment approach, the cell culture medium was simultaneously supplemented with ATRA and H_2_O_2_ for a 7-day period, while in the pre-treatment regimen, cells were first exposed to H_2_O_2_ for 72 h and, upon its removal, treated with ATRA for 7 days. All experiments were performed in biological triplicates.

### 2.3. RNA Extraction and Integrity Analysis

RNA was extracted from differentiated cells, as explained before [12]. Briefly, having lysed cells with 1 mL Trizol reagent (Sigma-Aldrich, St. Louis, MO, USA) and centrifuged with 200 µL chloroform (Chem-supply, Gillman, SA, Australia), total RNA was trapped in an aqueous phase and precipitated by adding 80 μg glycogen (Life Technologies, Mulgrave, VIC, Australia) and 500 μL isopropanol (Chem-supply, Gillman, SA, Australia), followed by an overnight incubation at −20 °C. RNA was finally dissolved in nuclease-free water, and its integrity and concentration were checked by the Agilent small RNA kit and the 2100 Bioanalyzer according to the manufacturer’s instructions (Agilent Technologies, Santa Clara, CA, USA). All samples had RNA integrity number (RIN) values above 8.5.

### 2.4. Small RNA-Sequencing

QIAseq miRNA Library Kit and Illumina NextSeq 500 platform were used for small RNA library preparation and sequencing, respectively, by the Ramaciotti Centre for Genomics (UNSW, Sydney, NSW, Australia).

### 2.5. Processing of Sequencing Data and Differential Expression Analysis

FastQC (v0.11.8) (http://www.bioinformatics.babraham.ac.uk/projects/fastqc) (accessed on 5 February 2021) was implemented to check the quality of the sequencing FASTQ files. The low-quality nucleotides (Phred quality score < 28) as well as sequencing adapters were then removed by Cutadapt (v2.10) (https://cutadapt.readthedocs.io/en/v2.10/installation.html) (accessed on 5 February 2021). Having mapped to the human genome build hg19 using Bowtie2 (v2.4.1) [19], the aligned reads were annotated to miRNAs and quantified with htseq-count (v0.7.2) [20].

Read counts were normalised to sequencing depth (counts per million, CPM) using edgeR (v3.6.1) [21]. A CPM threshold was then employed to remove miRNAs consistently expressed to a very low degree across samples (5 raw counts in the smallest library). A final analysis based on the pairwise exact test of treated versus control samples returned a list of differentially expressed miRNAs, with Benjamini–Hochberg false discovery rate (FDR) below 0.05 and absolute log2 fold change (|log2FC|) above 0.6 considered significant.

### 2.6. Identification of Psychiatry-Related Dysregulated miRNAs

We next checked if any of the miRNAs with altered expression in our experiments were previously reported to be differentially expressed in psychiatric patients compared to healthy controls. To do that, we referred to a recently published systematic review by Smigielski et al. [17], which summarised the findings of 42 studies on postmortem brains and biofluids obtained from psychiatric individuals, mostly schizophrenia patients, and non-psychiatric controls. In order to eliminate the inconsistency between miRNA IDs reported by previous studies, we used the online tool miRNAmeConverter (https://bioconductor.org/packages/release/bioc/html/miRNAmeConverter.html) [22] (accessed on 12 March 2021) to convert our differentially expressed miRNA IDs to version 17 of miRBase [23]. A one-sided Fisher’s exact test was applied to examine the enrichment of miRNAs responsive to oxidative stress in psychiatric diseases.

### 2.7. miRNA-mRNA Correlation Analysis and Network Visualisation

In order to identify experimentally supported interactions between miRNAs and their TargetScan-predicted mRNA targets [24], we integrated current results with those from total RNA-sequencing [12] through a customised script that calculated the Pearson’s correlation coefficient between miRNA-mRNA pairs that were differentially expressed in our experiments. The pairs that were negatively (correlation coefficient < 0) and significantly (FDR < 0.05) correlated were then visualised using the Cytoscape software platform v.3.7.1 [25].

### 2.8. Gene Set Enrichment Analysis (GSEA)

The functional implications of differentially expressed miRNAs were determined by investigating the enrichment of their negatively correlated dysregulated mRNA targets in various gene ontology terms using the ToppGene functional enrichment suite [26].

## 3. Results

### 3.1. Confirmation of Differentiation

In this study, we used two distinct treatment regimens. In the pre-treatment approach, SH-SY5Y cells were exposed to oxidative stress for 3 days prior to differentiation. Alternatively, in the co-treatment approach, the cells were exposed to oxidative stress during the 7 days of differentiation. As explained in detail previously [12], both experiments led to the generation of differentiated neuron-like cells with typical neurite outgrowth and expression of neuronal marker genes, namely MAPT, ENO2, TUBB3, and SV2A.

### 3.2. miRNA Expression in Response to Oxidative Stress

**Figure 1:**
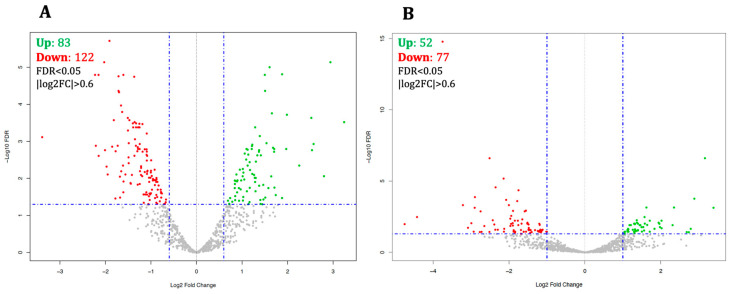
Volcano plot of peroxide treatment-associated miRNA expression. (**A**) Seven days of treating SH-SY5Y neuroblastoma cells with H_2_O_2_ and ATRA simultaneously resulted in the increased and decreased expression of 83 and 122 miRNAs, respectively, whereas (**B**) a three-day pre-treatment of cells with H_2_O_2_ before adding ATRA caused expression alteration of 129 miRNAs, 52 up- and 77 down-regulated, in comparison to the cells differentiated in the absence of peroxide (FDR < 0.05 (horizontal blue line) and |log2FC| > 0.6 (vertical blue lines)). The red and green dots represent down- and up-regulated miRNAs, respectively.

**Figure 2:**
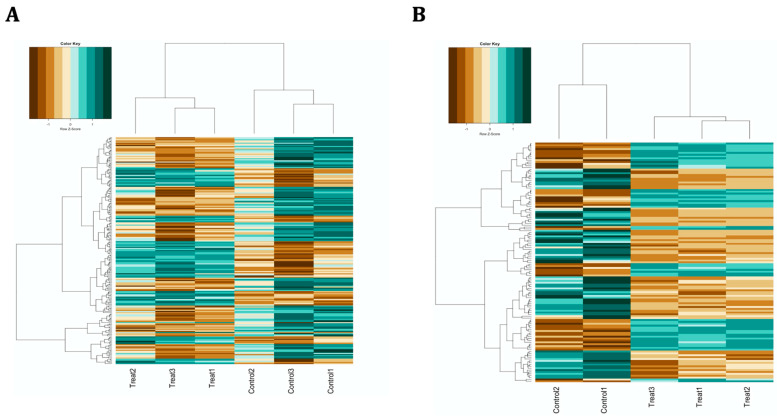
Unsupervised hierarchical clustering of the differentially expressed miRNAs in (**A**) co-treatment and (**B**) pre-treatment conditions. The heatmap categorises samples based on relatedness degree in the expression patterns of their differentially expressed miRNAs, with each column and row representing a unique sample and a unique miRNA, respectively. Control and treatment samples were clearly distinguishable in both experiments.

A differential expression analysis revealed substantial changes in the expression of miRNAs in both experiments compared to cells differentiated in the absence of oxidative stress, with a maximum threshold of 0.05 for false discovery rate (FDR) and a minimum threshold of 0.6 for |log2FC|. As to the co-treatment condition, 205 miRNAs were differentially expressed, including 122 down-regulated and 83 up-regulated miRNAs (Figure 1A and Supplementary Table S1), whereas in the pre-treatment condition, 77 and 52 miRNAs showed decreased and increased expression, respectively, for a total of 129 dysregulated miRNAs (Figure 1B and Supplementary Table S2). As revealed by unsupervised hierarchical clustering, the control and treated samples were clearly segregated by the pattern of differentially expressed genes (Figure 2).

A closer examination of the molecules differentially expressed in each treatment revealed that most were distinct to each condition. While 33 were common to both the pre- and co-differentiation regimens, all except for 5 showed the opposite direction of effect, including hsa-miR-34b-3p, hsa-miR-34c-5p, hsa-miR-432-5p, hsa-miR-488-5p, and hsa-miR-664a-3p (Supplementary Table S3).

### 3.3. Oxidative Stress-Associated miRNAs Related to Psychiatric Disorders

**Figure 3:**
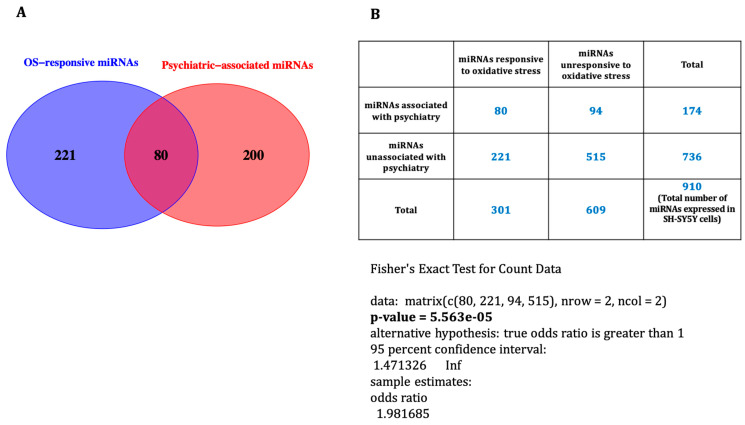
(**A**) A total of 80 miRNAs responsive to oxidative stress were previously reported to be differentially expressed in psychiatric disorders. (**B**) Fisher’s exact test revealed that the oxidative stress-responsive miRNAs were significantly enriched in psychiatric diseases.

To screen for prior association with psychiatric disorders, we cross-referenced our observations with a list of 280 psychiatry-associated miRNAs collated by Smigielski et al. in their systematic review [17]. This analysis revealed that 23% (*n* = 48) and 34% (*n* = 44) of miRNAs for co- and pre-treatment, respectively, had previously been observed to be dysregulated in psychiatric disorders (Supplementary Tables S1 and S2). Combining the two treatments, the results showed that, of the 301 differentially expressed miRNAs, 80 were associated with psychiatric diseases (Figure 3A), and we observed significant enrichment of oxidative stress-responsive miRNAs in psychiatric disorders using Fisher’s exact test (*p*-value = 5.563 × 10^−5^) (Figure 3B).

This included 12 psychiatry-related miRNAs that were affected by both experiments, namely hsa-miR-101-3p, hsa-miR-130a-3p, hsa-miR-15a-5p, hsa-miR-21-5p, hsa-miR-26b-5p, hsa-miR-301a-3p, hsa-miR-34c-5p, hsa-miR-340-5p, hsa-miR-432-5p, hsa-miR-483-3p, hsa-miR-664a-3p, and hsa-miR-99b-5p. While most of these molecules have also been reported in multiple studies (Table 1), it should be noted that several reports of psychiatry-related miRNAs did not meet the inclusion criteria for systematic review by Smigielski et al. [17].

### 3.4. miRNA-mRNA Expression Correlation and Network Construction

**Figure 4:**
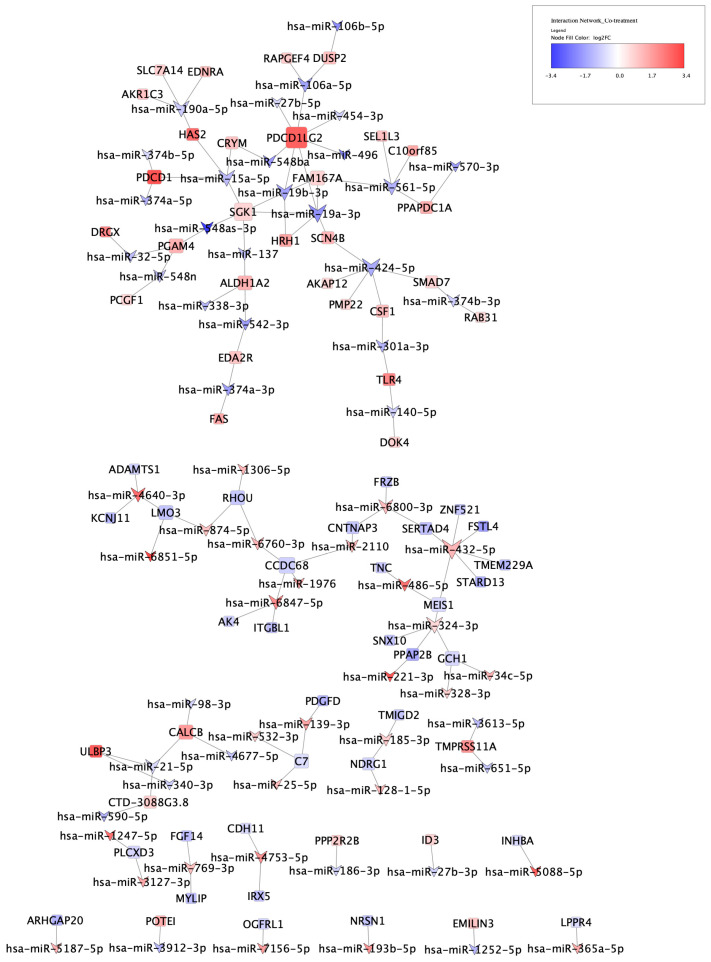
miRNA-mRNA interaction network for co-treatment condition. Comparing the list of differentially expressed miRNAs (*n* = 205) and mRNAs (*n* = 295) with the miRNA-mRNA interactions predicted by TargetScan returned 121 negatively correlated pairs, depicted by graph edges (FDR < 0.05), constituting a network of 65 miRNAs and 70 mRNAs. V shapes and rectangles represent miRNAs and mRNAs, respectively, and blue and red depict down- and up-regulation, respectively. The nodes’ colour intensity is proportional to log2FC, while the nodes’ size is proportional to their number of interactions.

To explore the relationship between oxidative stress-associated miRNA and the predicted target genes, we examined the correlation between the current miRNA data and the previously published mRNA expression from the same samples [12]. This revealed that for the co-treatment condition, 121 TargetScan-predicted miRNA-mRNA pairs were negatively correlated with each other, with correlation coefficients between −0.99 and −0.92 and FDR < 0.05 (Supplementary Table S4), suggesting that 65 unique miRNAs were directly regulating the expression of 70 unique mRNAs. The structure of the putative interaction network was graphed using Cytoscape (Figure 4).

**Figure 5:**
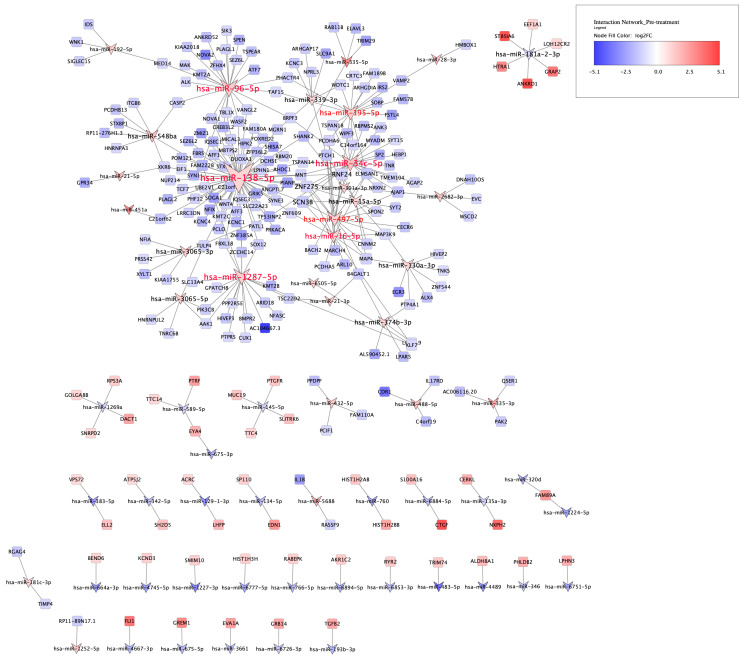
miRNA-mRNA interaction network for pre-treatment condition. Of the 129 differentially expressed miRNAs and 3791 differentially expressed mRNAs, 59 miRNAs and 233 mRNAs were significantly correlated with each other, forming a network of 312 negative interactions represented by graph edges (FDR < 0.05). V shapes and rectangles represent miRNAs and mRNAs, respectively, and blue and red depict down- and up-regulation, respectively. The nodes’ colour intensity is proportional to log2FC, while the size of the nodes and their labels are proportional to the number of their interactions. Nodes with red labels are the 7 most connected miRNAs, accounting for 56% of the network interactions.

By comparison, in the pre-treatment condition, 59 unique miRNAs and 233 unique mRNAs were significantly and negatively correlated through 312 direct interactions (−0.99 < correlation coefficient < −0.97 and FDR < 0.05) (Supplementary Table S5 and Figure 5).

An interesting observation in the pre-treatment condition was that seven miRNAs, all up-regulated, comprised 56% (177/312) of the network interactions, such that more than half of mRNAs, 134 out of 233, were directly regulated by these seven miRNAs, and all showed decreased expression. These miRNAs (and their number of direct targets) include hsa-miR-138-5p (*n* = 66), hsa-miR-96-5p (*n* = 24), hsa-miR-34c-5p (*n* = 21), hsa-miR-1287-5p (*n* = 20), hsa-miR-497-5p (*n* = 16), hsa-miR-195-5p (*n* = 16), and hsa-miR-16-5p (*n* = 14). Interestingly, all these miRNAs except hsa-miR-1287-5p were reported in previous studies to be dysregulated in tissues from people with psychiatric disorders compared to those from healthy controls (Supplementary Table S2).

### 3.5. Functional Enrichment Analysis

**Figure 6:**
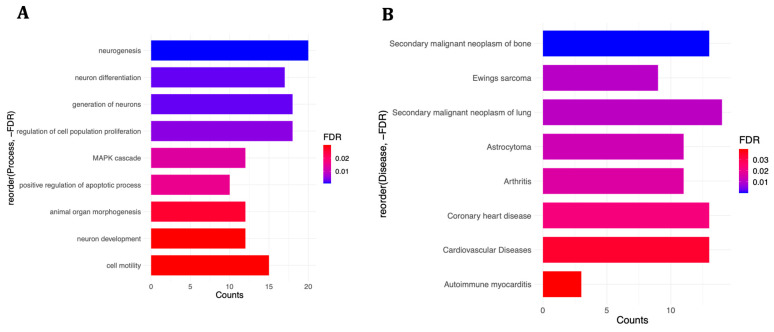
Enrichment analysis for 69 differentially expressed mRNAs in SH-SY5Y cells negatively correlated with differentially expressed miRNAs following simultaneous treatment with ATRA and H_2_O_2_. (**A**,**B**) illustrate enriched biological processes and diseases, respectively. For a complete detailed list, see Supplementary Table S6.

In order to understand the functional implications of differentially expressed miRNAs, the enrichment of their negatively correlated mRNA pairs in various biological processes, cellular components, and diseases was investigated using ToppFun. As to the co-treatment condition, the 69 dysregulated genes were involved mostly in neurobiology-related processes, such as neurogenesis (20 genes), neuron differentiation (17 genes), and neuron development (12 genes) (Figure 6A), although they were not significantly localised in any cellular spaces. A variety of malignancies, cardiovascular diseases, and inflammatory or immune disorders were among the enriched diseases (Figure 6B). The detailed list of enriched categories and their corresponding gene sets can be found in Supplementary Table S6.

**Figure 7:**
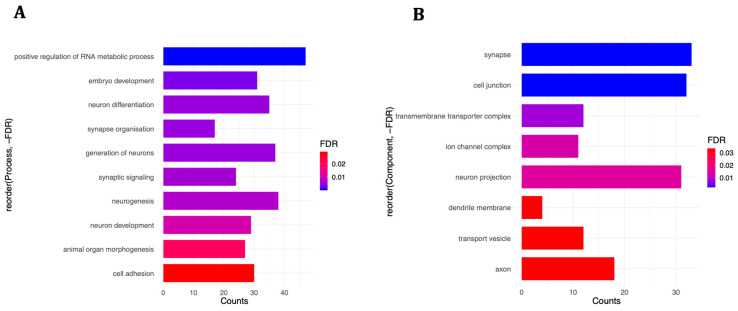
Selected enriched (**A**) biological processes and (**B**) cellular components among the 233 mRNAs involved in the miRNA-mRNA interaction network of the pre-treatment condition. For a complete detailed list, see Supplementary Table S7.

The 233 mRNAs negatively correlated with the pre-treatment-affected miRNAs were enriched in similar neurobiology-related processes as well as synapse organisation (17 genes) and synaptic signalling (24 genes) (Figure 7A). These were mostly localised in the synapse (33 genes) and cell junction (32 genes) (Figure 7B), and, interestingly, they were involved in two neurodevelopmental disorders: intellectual disability (ID) (35 genes) and global developmental delay (GDD) (19 genes). Supplementary Table S7 lists the statistically significant enriched categories and their corresponding gene sets. Interestingly, when we focused on the top 7 most connected miRNAs, with at least 10 connections, and their 134 negatively correlated mRNAs (down-regulated), they also returned very similar ontologies, including neuron differentiation (23 genes), synapse organisation (13 genes), synaptic signalling (17 genes), and intellectual disability (22 genes) (Supplementary Table S8). By contrast, the remaining 99 mRNAs were not enriched in any relevant categories. This suggests that the functional impact of the miRNA-mRNA interaction network in the pre-treatment experiment is coordinated by the seven miRNA nodes or hubs with the highest number of interactions.

## 4. Discussion

In this study, we tested the hypothesis that the observed widespread changes in gene expression that were induced by oxidative stress in differentiating human neuroblasts [12] are associated with dysregulation of miRNA. In accordance with expectations, these trans-acting stress-responsive signalling molecules [18] were differentially expressed under both the co-treatment (205 miRNAs) and pre-treatment (129 miRNAs) conditions. Interestingly, a substantial proportion of miRNAs impacted by oxidative stress exposure were previously reported to be dysregulated in psychiatric disorders, mostly schizophrenia, as reviewed by Smigielski et al. [17] and referenced here in Table 1 and Supplementary Tables S1 and S2. In the following paragraphs, we discuss selected miRNAs that were affected by oxidative stress exposure and implicated in neurodevelopment and psychiatric disorders.

As to the co-treatment condition, an interesting observation in the miRNA-mRNA network was the direct interaction of ALDH1A2 with three schizophrenia-related miRNAs: hsa-miR-137, hsa-miR-542-3p, and hsa-miR-338-3p (Figure 4). As discussed in detail before [12], genetic studies indicate an association between ALDH1A2 and some other retinoic acid signalling genes and psychiatric disorders. Also, expression dysregulation of hsa-miR-338-3p [27,28] and hsa-miR-542-3p [29] has been reported in some brain regions of patients with schizophrenia. hsa-miR-137, on the other hand, is a brain-enriched miRNA involved in the regulation of various neurodevelopmental processes, such as neurogenesis, neuronal maturation, and the development of dendrites [30]. A single nucleotide polymorphism (SNP) in proximity to the MIR137 gene was identified as the second most significantly associated variant with schizophrenia risk in the largest schizophrenia genome-wide association study (GWAS) [31]. In addition, we reported an SNP [32] and a variable-number tandem repeat (VNTR) [33] in this miRNA gene to be associated with cognitive impairment in patients with schizophrenia. Although increased expression of miR-137 in peripheral tissues from patients with schizophrenia has been reported by several studies [34,35,36], there exists no report of its dysregulation in postmortem brains. However, Arakawa et al. recently observed schizophrenia-associated behavioural deficits, including social and cognitive deficits, in transgenic mice with overexpression of miR-137 in the whole brain [37].

Another well-studied miRNA with implications for brain development and psychiatry that was affected in the co-treatment experiment was hsa-miR-17-5p. It is a member of the miR-17 family, which has significantly higher expression levels during the early stages of corticogenesis. hsa-miR-17-5p, for example, is expressed 20 times more in embryonic day E12.5 compared to postnatal day P60 in the mouse cortex and has a critical role in regulating the proliferation and differentiation of neural precursor cells to neurons and glial cells [38]. Consistently, we observed down-regulation of the entire miR-17 family, including miR-17-5p, following retinoic acid-induced differentiation of SH-SY5Y neuroblasts [39]. Expression dysregulation of this miRNA has been reported by several studies in different brain regions as well as peripheral tissues of patients with schizophrenia and/or schizoaffective disorder (SZAD) compared to non-psychiatric controls (Table 1). In accordance with our observations, miR-17-5p was reported as an oxidative stress-responsive miRNA by Chen et al. In a rat model of neonatal hypoxia–ischemia (HI), which causes brain injury and long-term behavioural and cognitive deficits, they observed that endoplasmic reticulum (ER) stress, induced by oxidative stress, triggered degeneration of miR-17-5p, which ultimately resulted in inflammatory activation and brain injury [40].

In the pre-treatment condition, the most affected miRNA was hsa-miR-223-3p, with a 26-fold expression decrease. It is a microglia-enriched [41], exosome-secreted miRNA [42] suggested to be involved in neurodevelopment and psychiatric disorders, with most of its predicted targets expressed in the brain [43]. Up-regulation of this miRNA, among many others, was observed following in vitro induction of differentiation in human neural stem/progenitor cells (NS/PCs). Through further functional experiments, the authors suggested miR-223-3p as a regulator of several neuronal features, such as cell body size, dendrite total length, and neuronal activity [43].

Our group was the first to report increased expression of this miRNA in the dorsolateral prefrontal cortex (DLPFC) of schizophrenia cases [27]. Zhao et al. later observed a similar trend in plasma samples from patients with first-episode schizophrenia (FES) [44]. They could experimentally validate the direct interaction of this miRNA with four of its predicted targets, which are implicated in neuronal development and migration, as well as schizophrenia pathophysiology, namely SYNE1, SKIL, RHOB, and INPP5B [44]. More recently, elevated expression of miR-223-3p was also reported in the orbitofrontal cortex (OFC) of patients with schizophrenia and BD [42]. Interestingly, and consistent with our current observation of this miRNA’s strong responsiveness to oxidative stress, Harraz et al. showed its neuroprotective function in response to neuronal injury, for example in stroke, transient global ischemia, and neurodegenerative disorders, by regulating the expression of two glutamate receptor genes, NR2B (GRIN2B) and GLUR2 (GRIA2) [45].

Another striking miRNA, hsa-miR-138-5p, had the highest number of negative correlations in the miRNA-mRNA interaction network for the pre-treatment condition, with 66 direct targets constituting around 20% of network interactions (Figure 5). It is a brain-enriched miRNA localised in dendrites, or, more precisely, the synaptodendritic compartment, that negatively regulates the growth and morphogenesis of dendritic spines in excitatory synapses. It is also thought to be involved in the regulation of synaptic plasticity [46]. Notably, dendritic spine abnormalities are among the most commonly observed neuropathological changes in postmortem brain tissues in patients with schizophrenia [47]. Interestingly, we observed increased expression of hsa-miR-138 in the superior temporal gyrus (STG) of schizophrenia cases [27], while Moreau et al. reported its down-regulation in the prefrontal cortex (PFC) of people with schizophrenia [28]. This miRNA was observed to be down-regulated in the hippocampus of memory-impaired aged mice, with higher levels being correlated with better memory performance in the novel object recognition task [48]. Consistent with this observation, a GWAS on 13 memory traits revealed an association between an SNP in a putative regulatory region of hsa-miR-138-5p (rs9882688) and memory performance in individuals aged 60 years and older [49].

Two molecules altered during pre-treatment oxidative stress, hsa-miR-195-5p and hsa-miR-181b-5p, are the most recurrently reported miRNAs dysregulated in psychiatric disorders (Table 1). miR-195-5p, which is implicated in many pathologies, including cancer [50], has a developmental-specific expression pattern in the human PFC [51], and its expression is regulated by the redox-sensitive transcription factor NF-kB [52,53,54]. miR-195-5p was observed to be associated with the enhancement of proliferation and the repression of neuronal and astrocyte differentiation of adult neural stem cells (aNSCs) derived from the dentate gyrus (DG) of the adult hippocampus, one of the few brain regions where neurogenesis persists after completion of embryonic development [55]. Intriguingly, some studies suggest a link between this miRNA and cognitive function. For example, Ai et al. demonstrated that the memory impairment and dementia induced by chronic brain hypoperfusion (CBH) could be prevented in rats through miR-195-5p overexpression and the resultant suppression of two of its putative targets, APP and BACE1, both of which are up-regulated during CBH and are associated with amyloid-β (Aβ) aggregation and cognitive impairment [54]. Similarly, Cao et al. recently showed that cognitive deficits associated with the apolipoprotein E4 (ApoE4) allele, a risk factor for Alzheimer’s disease (AD), could be rescued in mice by overexpression of miR-195 [56].

miR-181b-5p, on the other hand, belongs to the miR-181 family, which is a group of known regulators of key biological processes such as proliferation, apoptosis, embryonic development, and mitochondrial function involved in various neurodegenerative disorders and malignancies, as reviewed by Indrieri et al. [57]. In particular, miR-181b-5p is implicated in the pathology of the brain cancers astrocytoma and glioblastoma [57]. Up-regulation of this miRNA is one of the most consistent observations in patients with schizophrenia [17]. It was first reported by our group in the STG [58] and DLPFC [27] of postmortem brains and later replicated in peripheral tissues, including plasma, serum, and whole blood (Table 1). Notably and consistent with our current results, Casey et al. observed rapidly increased expression of miR-181b in the arterial blood of a piglet model of neonatal hypoxic–ischemic encephalopathy (HIE) at one hour post-HI, which persisted for 72 h [59].

In accordance with the previously reported changes in mRNA expression [12], there was little overlap between the two treatment regimens, such that only 33 miRNAs were common to both conditions. Most of these (*n* = 28) were inconsistent in terms of direction change. hsa-miR-432-5p was one of the five miRNAs affected and showed the same direction of expression change, i.e., up-regulation, in both treatments. It was also the most connected miRNA in the miRNA-mRNA interaction network of the co-treatment condition, directly targeting six mRNAs (Figure 4). Lai et al. were the first to report decreased expression of hsa-miR-432-5p in mononuclear leukocytes in patients with schizophrenia [60]. Later, we [61], and more recently, Yu et al. [62] also observed a reduction in its expression in peripheral blood mononuclear cells (PBMCs) of cases with schizophrenia. This miRNA, along with 11 other oxidative stress-induced differentially expressed miRNAs, originates from the same genomic location within the DLK1-DIO3 region on the long arm of chromosome 14 (14q32). The locus encodes 54 miRNAs residing in two large neighbouring clusters at 14q32.2 and 14q32.31, harbouring 10 and 44 miRNA genes, respectively [63]. Our group first reported substantial down-regulation of 17 miRNAs from this region in the PBMCs of 112 patients with schizophrenia compared to 76 non-psychiatric controls in a microarray analysis. By including molecules that were not significantly dysregulated after multiple testing correction, a further 10 miRNAs from the same region showed a similar trend of expression reduction in patients. A copy number variation analysis in a subset of samples revealed that the expression decrease was not due to structural variations in the locus. Remarkably, a pathway analysis of genes predicted by both the miRanda and TargetScan algorithms to be targeted by the 17 dysregulated miRNAs demonstrated their involvement in neural connectivity and synaptic plasticity [61]. We later observed changes in the expression of DLK1-DIO3 miRNAs in the left hemisphere of the entorhinal cortex (EC), an important brain region for high-level cognitive function implicated in schizophrenia, in a mouse model of maternal immune activation and adolescent cannabinoid exposure, two important risk factors for schizophrenia. This included differential expression of 25 miRNAs, although 5 were not statistically significant after correction for multiple testing following treatment with the viral mimic poly I:C alone, the synthetic cannabinoid HU210 alone, or a combination of them [64]. More strikingly, a recent study by Baulina et al. [63] reported up-regulation of 26 miRNAs from this region in the PBMCs of eight treatment-naive male patients with relapsing–remitting multiple sclerosis (RRMS) compared to four healthy controls, but not in a female cohort of the same size [65], which is an interesting observation considering the shared genetic risk between schizophrenia and MS [66] and also the higher rate of psychiatric disorders among MS patients in comparison to the general population [67,68,69].

Comparing our results with the above-discussed three studies [61,63,64] shows a noticeable overlap, so that 9 out of 12 DLK1-DIO3 miRNAs affected following exposure to oxidative stress were also demonstrated to be dysregulated in at least one of those studies (Table 2).

Of particular interest are hsa-miR-134-5p and hsa-miR-370-3p, which were significantly differentially expressed across all three investigations. hsa-miR-134-5p is a brain-enriched miRNA implicated in synaptic development and plasticity through negative regulation of dendritic spine volume, as shown by its gradual increase in the synaptodendritic compartment of rat hippocampal neurons during brain development, reaching its maximum level at postnatal day P13, when synaptic maturation occurs [70]. Schizophrenia-associated changes in this miRNA expression have been repeatedly reported (Table 1). We first observed its increased expression in the dorsolateral prefrontal cortex (DLPFC) Brodmann Area 46 (BA46) samples from 37 matched pairs of schizophrenia/schizoaffective disorder (SZAD) cases and non-psychiatric controls [29] and later showed that it was down-regulated in the PBMCs of patients with schizophrenia [61]. Decreased expression of miR-134-5p in the PBMCs of subjects with schizophrenia was more recently confirmed and suggested as a diagnostic biomarker in another independent study by Yu et al. [62]. A similar trend of this miRNA reduced expression has been observed in the plasma of patients with BD [71] and MDD [72], as well as in the plasma, hippocampus, and PFC in a rat model of depression [72]. hsa-miR-370-3p, which is suggested to contribute to mouse embryonic development, especially brain morphogenesis [73], is also differentially expressed in psychiatric diseases. While we observed down-regulation of this miRNA in the PBMCs of patients with schizophrenia [61], Lee et al. reported its increased expression in serum samples from subjects with bipolar disorder compared with controls [74]. Also, it was significantly down-regulated in the hippocampus in a rat model of depression [75].

Disease-associated changes in the expression level of some of the above-discussed miRNAs show the same trend, up- or down-regulation, in the brain and peripheral tissues. However, it should be noted that miRNA levels in peripheral tissues might not directly reflect their expression levels in brain tissue, unless there is experimental data supporting such an association.

Combining miRNA and mRNA datasets showed that a subset of differentially expressed genes with implications for neurodevelopmental processes and disorders were directly regulated by differentially expressed miRNAs in both experiments, especially the pre-treatment condition (Figure 6 and Figure 7). In addition, some miRNA-targeted genes in the co-treatment experiment were enriched in cardiovascular diseases and coronary heart disease (Figure 6), which suggests that, apart from the nervous system, prenatal exposure to oxidative stress might adversely affect the cardiovascular system as well [76] and might explain part of the higher risk for cardiovascular diseases among patients with psychiatric disorders [77]. Interestingly, some of the DLK1-DIO3 region miRNAs are known to be differentially expressed in cardiovascular development and/or diseases, as comprehensively reviewed by Dill and Naya [78], and are also affected by our oxidative stress treatments. For instance, miR-432, miR-370, and miR-495 have been associated with atrial fibrillation [79], coronary artery disease [80], and cardiac fibrosis [81], respectively. miR-134, on the other hand, was demonstrated as a modulator for the proliferation of human cardiomyocyte progenitor cells, which play critical roles in the early development of the heart [82], and a significant increase in its plasma levels was suggested as a diagnostic biomarker for acute pulmonary embolism [83].

The enrichment of immunity-related disorders was another remarkable observation in the co-treatment condition (Figure 6), given the well-established link between immune system dysfunction and psychiatric disorders, which was comprehensively discussed in our previous paper [12]. Our results from these two transcriptome studies support the inflammatory mediator hypothesis, suggesting that the inflammatory immune system might mediate, at least in part, the association between stress and psychiatric disorders [84,85] and, even more strikingly, act as a linker among stress, psychiatric disorders, and cardiovascular diseases, which might explain part of the high comorbidity between these illnesses and their association with early-life stress [86,87,88].

Our study has some limitations that need to be considered when interpreting the results. The cell model we used, the SH-SY5Y cell line, is the most cited in vitro model for neuropsychiatric research because of its remarkable advantages, such as feasibility and low cost to culture, large-scale expandability prior to differentiation, literature availability, reproducibility, and the absence of ethical concerns [89]. However, it also has limitations. The SH-SY5Y cell line is a subline of the SK-N-SH cell line, derived from a female patient with neuroblastoma. Many studies have shown that the sex of cells affects their proliferation, differentiation, and stress response [90], and therefore, our adopted model cannot address sex differences. In addition, the parental SK-N-SH cell line comprises not only neuroblast-like cells but also epithelial-like cells. And although the SH-SY5Y cell line was developed as a homogenous neuroblastic clone, epithelial cells still exist in the cell culture. Since the postmitotic effects of retinoic acid do not affect epithelial-like cells, they keep proliferating and constitute an important percentage of cells in the resultant neuronal culture [91]. Also, due to its tumour origin, the SH-SY5Y cell line has genetic peculiarities that affect its growth performance, viability, differentiation fate, and response to stress [92]. Designing similar studies using primary cell models, such as embryonic stem cells and induced pluripotent stem cells (iPSCs), can address most of these limitations and provide further support for the findings of the current study.

On the other hand, our transcriptome analysis approach addresses one aspect of miRNA-mediated gene expression repression, i.e., degrading mRNA transcripts. However, miRNAs are known to suppress gene expression through translational attenuation as well. Future proteomics studies can capture this mechanism of miRNA action and provide a broader picture of the neuronal cell response to oxidative stress.

It should also be noted that many miRNAs are developmental-specific, and their expression changes during differentiation of SH-SY5Y cells [93]. The current study has only focused on miRNA expression in mature, differentiated neurons to exclusively investigate the impact of oxidative stress on miRNA expression. Still, some inferences can be made about the effect of the neuronal differentiation process.

## 5. Conclusions

Collectively, we show that chronic exposure to oxidative stress before or during the differentiation process, even at very low, non-cytotoxic levels, causes widespread changes in the expression of miRNAs, a well-known class of gene expression regulatory molecules. Many of the dysregulated miRNAs are associated with psychiatric disorders, and their predicted target genes are enriched in synapses and critical neurodevelopmental processes. This suggests that despite outwardly appearing as fully developed neurons, the underlying molecular frameworks may be compromised, affecting nervous system development and connectivity. As a result, this level of exposure to oxidative stress may potentially increase the probability of developing psychiatric disorders later in life. Understanding these molecular intricacies is crucial for elucidating the pathophysiology of psychiatric disorders and developing targeted interventions to reduce the risk.

## Figures and Tables

**Table 1:** Oxidative stress-associated miRNAs also reported to be dysregulated in psychiatric disorders by more than two independent studies. SZ: schizophrenia; SZAD: schizophrenia and/or schizoaffective disorder; STG: superior temporal gyrus; DLPFC: dorsolateral prefrontal cortex; PFC: prefrontal cortex; PBMCs: peripheral blood mononuclear cells.

Condition	miRNA ID		Direction of Change	Previous Reports			
	New	Old		Diagnosis	Tissue	Direction of Change	Reference (in Smigielski et al. [17])
**Co-treatment**	hsa-miR-17-5p	hsa-miR-17	Down	SZ, SZAD	STG, DLPFC, PFC, serum	Up (brain) and down (serum)	103, 184, 185, 186, 195
	**hsa-miR-432-5p**	hsa-miR-432	Up	SZ	PBMCs, leukocyte	Down	191, 192, 204
	hsa-miR-106b-5p	hsa-miR-106b	Down	SZ	PFC	Up and down	179, 180, 185
	hsa-miR-30b-5p	hsa-miR-30b	Down	SZ, SZAD	PFC, STG	Up and down	178, 180, 181
	hsa-miR-29c-3p	hsa-miR-29c	Down	SZ, SZAD	PFC exosomes, DLPFC, PFC	Up and down	103, 170, 180
	hsa-miR-328-3p	hsa-miR-328	Up	SZ, SZAD	STG, DLPFC	Up	103, 181, 184
	hsa-miR-652-3p	hsa-miR-652	Down	SZ, SZAD	DLPFC, leukocyte, plasma	Up	184, 192, 199
	hsa-miR-33a-5p	hsa-miR-33	Down	SZ	PFC exosomes, PFC, DLPFC	Up and down	103, 170, 179
**Pre-treatment**	hsa-miR-195-5p	hsa-miR-195	Up	SZ, SZAD	STG, PFC, plasma, whole blood, serum, PBMCs	Up and down	103, 180, 182, 188, 194, 195, 198
	hsa-miR-181b-5p	hsa-miR-181b	Up	SZ	STG, DLPFC, whole blood, serum, plasma	Up	102, 103, 194, 195, 196, 197
	hsa-miR-107	hsa-miR-107	Up	SZ, SZAD	STG, PFC, DLPFC, PBMCs	Up and down	103, 123, 184, 191
	**hsa-miR-432-5p**	hsa-miR-432	Up	SZ	PBMCs, leukocyte	Down	191, 192, 204
	hsa-miR-193b-3p	hsa-miR-193b	Down	SZ	PFC, plasma, whole blood	Up and down	179, 199, 205
	hsa-miR-134-5p	hsa-miR-134	Down	SZ, SZAD	DLPFC, PBMCs	Up and down	184, 191, 204
	hsa-miR-409-3p	hsa-miR-409-3p	Down	SZ	STG, DLPFC, PBMCs, whole blood	Up and down	103, 191, 205
	hsa-miR-346	hsa-miR-346	Down	SZ	DLPFC, serum, plasma	Up and down	187, 195, 197

Bold miRNAs are affected by both treatments. The reference numbers listed here are the original numbers reported by Smigielski et al. in their recent systematic review [17].

**Table 2:** List of differentially expressed miRNAs from DLK1-DIO3 locus following exposure of SH-SY5Y cells to oxidative stress and their association with schizophrenia (SZ) [61], MS [63], and maternal immune activation [64]. The check marks indicate the miRNAs that were reported by each study.

miRNA	Log2FC	Gardiner et al. [61]		Hollins et al. [63]		Baulina et al. [64]
		Significantly Down-Regulated in SZ after Multiple Testing Correction	Average Expression Reduction in SZ, but Insignificant after Multiple Testing Correction	Significant DE in the Left Hemisphere after Multiple Testing Correction	DE in the Left Hemisphere, But Insignificant after Multiple Testing Correction	Significantly Up-Regulated in Male Patients with MS after Multiple Testing Correction
**Co-treatment**						
miR-432-5p	1.3	✓				✓
miR-370-3p	1.2	✓		✓		✓
miR-485-3p	1	✓			✓	✓
miR-495-3p	−1.6		✓	✓		✓
miR-376b-3p	−1.4		✓	✓		✓
miR-889-3p	−0.88		✓			✓
miR-758-3p	−1.7			✓		✓
miR-655-3p	−2					
miR-496	−2					
miR-369-3p	−0.8					
**Pre-Treatment**						
miR-432-5p	1.3	✓				✓
miR-323a-3p	−1.5	✓		✓		
miR-134-5p	−1.8	✓		✓		✓
miR-485-5p	−1.9	✓			✓	✓

## Data Availability

The data presented in this study, including raw sequencing and processed read count data, are openly available in the Gene Expression Omnibus (Accession Number: GSE182627).

## Supplementary Materials

The following supporting information can be downloaded at https://www.mdpi.com/article/10.3390/life14050562/s1: Table S1: List of dysregulated miRNAs in co-treatment experiment (FDR < 0.05, |log2FC| > 0.6); Table S2: List of dysregulated miRNAs in pre-treatment experiment (FDR < 0.05, |log2FC| > 0.6); Table S3: List of dysregulated miRNAs in both experiments; Table S4: Statistically significant miRNA-mRNA negative correlations for co-treatment condition; Table S5: Statistically significant miRNA-mRNA negative correlations for pre-treatment condition; Table S6: Gene set enrichment analysis for co-treatment condition; Table S7: Gene set enrichment analysis for pre-treatment condition; Table S8: GSEA for 134 mRNAs targeted by the top 7 most connected miRNAs, i.e., ≥10 connections, in the pre-treatment condition, including miR-13.8-5p, miR-96-5p, miR-34c-5p, miR-1287-5p, miR-497-5p, miR-195-5p, and miR-16-5p.
